# Fluorine in the Blood

**Published:** 1857-10

**Authors:** 


*Fluorine in the Blood.*—The various researches which have, of late years, been made upon the blood, have not been unproductive of important results. Truth, indeed, is evolved slowly, but the patient research of the last ten or twenty years, has not been in vain. We are learning more and more, the fulness, so to speak, of the vital fluid. The solids appear to have less and less to do, as the progress of inquiry shows that the blood contains the elements of the secretions and excretions. Urea has long since been proved to exist in the blood before it was discharged by the urine. So the kidneys have been taken out of the category of *secreting*, and placed in those of *excreting* glands. The liver bids fair to follow, if the discovery of chloric acid pre-existent in the blood be substantiated.

Of the inorganic components of the body, all have been traced to the blood. One of these, however, fluorine, has long been a stumbling block to organic chemists. It has been found in the teeth, in the bones, and in the hair. Berzelius, who first discovered it in the bones, was led to believe that it was an accidental constituent derived from some extraneous source. Of late, however, its presence in those organs has been shown, by M. Jerome Nicklés, to be constant. He finds it to pervade the whole system, and finally traces it to its source in the blood. He even goes so far as to assert that it is essential to the health of the body, and that a species of chlorosis is induced by its absence, just as the same disease is brought about by a deficiency of iron.

